# Carnitine O-Acetyltransferase as a Central Player in Lipid and Branched-Chain Amino Acid Metabolism, Epigenetics, Cell Plasticity, and Organelle Function

**DOI:** 10.3390/biom15020216

**Published:** 2025-02-02

**Authors:** Mariateresa Volpicella, Maria Noemi Sgobba, Luna Laera, Anna Lucia Francavilla, Danila Imperia De Luca, Lorenzo Guerra, Ciro Leonardo Pierri, Anna De Grassi

**Affiliations:** 1 Department of Biosciences, Biotechnologies and Environment, University of Bari “Aldo Moro”; Via E. Orabona 4, 70125 Bari, Italy; mariateresa.volpicella@uniba.it (M.V.); maria.sgobba@uniba.it (M.N.S.); luna.laera@uniba.it (L.L.); anna.francavilla@uniba.it (A.L.F.); d.deluca21@studenti.uniba.it (D.I.D.L.); lorenzo.guerra1@uniba.it (L.G.); 2 Laboratory of Biochemistry, Structural and Molecular Biology, Department of Pharmacy—Pharmaceutical Sciences, University of Bari “Aldo Moro”, Via E. Orabona 4, 70125 Bari, Italy

**Keywords:** mitochondrial carnitine acetyltransferase, CRAT, peroxisomal carnitine octanoyltransferase, CROT, carnitine palmitoyltransferases, CPT, choline acyltransferase, ChAT, suramin, artemisinin, molecular modeling

## Abstract

Carnitine O-acetyltransferase (CRAT) is a key mitochondrial enzyme involved in maintaining metabolic homeostasis by mediating the reversible transfer of acetyl groups between acetyl-CoA and carnitine. This enzymatic activity ensures the optimal functioning of mitochondrial carbon flux by preventing acetyl-CoA accumulation, buffering metabolic flexibility, and regulating the balance between fatty acid and glucose oxidation. CRAT’s interplay with the mitochondrial carnitine shuttle, involving carnitine palmitoyltransferases (CPT1 and CPT2) and the carnitine carrier (SLC25A20), underscores its critical role in energy metabolism. Emerging evidence highlights the structural and functional diversity of CRAT and structurally related acetyltransferases across cellular compartments, illustrating their coordinated role in lipid metabolism, amino acid catabolism, and mitochondrial bioenergetics. Moreover, the structural insights into CRAT have paved the way for understanding its regulation and identifying potential modulators with therapeutic applications for diseases such as diabetes, mitochondrial disorders, and cancer. This review examines CRAT’s structural and functional aspects, its relationships with carnitine shuttle members and other carnitine acyltransferases, and its broader role in metabolic health and disease. The potential for targeting CRAT and its associated pathways offers promising avenues for therapeutic interventions aimed at restoring metabolic equilibrium and addressing metabolic dysfunction in disease states.

## 1. Introduction

Carnitine O-acetyltransferase (CRAT) is a mitochondrial enzyme that plays an indispensable role in maintaining cellular energy balance and metabolic flexibility [1,2,3,4,5]. By catalyzing the reversible transfer of acetyl groups between acetyl-coenzyme A (acetyl-CoA) and carnitine, CRAT plays a pivotal role in buffering excess mitochondrial acetyl-CoA generated when substrate oxidation surpasses energy demand [1,6]. Skeletal-muscle-specific CRAT knockout (KO) mice demonstrate the pathological consequences of mitochondrial acetyl-CoA accumulation, including inhibition of PDH activity [1,2,7], reduced oxygen consumption rates [1,8], and hyperacetylation of various mitochondrial proteins, notably subunits of PDH and respiratory chain complexes [1,9]

This buffering role should prevent acetyl-CoA accumulation that could otherwise inhibit critical metabolic processes and would allow cells to efficiently adapt to fluctuating energy demands, enabling the transition between fatty acid and glucose oxidation under varying metabolic conditions [1,2]. As part of the broader carnitine shuttle system, CRAT operates in conjunction with the mitochondrial carnitine/acylcarnitine carrier (SLC25A20) and carnitine palmitoyltransferases (CPT1 and CPT2), but also with the trifunctional protein and with enzymes of the β-oxidation, ensuring the coordinated transport and utilization of acyl groups across cellular compartments [1,2,3,4,5,10,11,12]. Remarkably, Acetyl-CoA, Acyl-CoA, and CoASH are essentially membrane impermeable. Indeed, they need dedicated antiporters of the SLC25A family for crossing mitochondrial inner membrane, with specific reference to the mitochondrial carnitine/acyl-carnitine antiporter (CAC, coded by SLC25A20 [13]), the mitochondrial carrier SLC25A42 [14,15,16] that facilitates the uptake of CoA in exchange with PAP, and the peroxisomal SLC25A17 antiporter [17,18] involved in the uptake of CoA, FAD, and NAD, in exchange for PAP, AMP, and FMN. In this regard, it is good to notice that cells possess limited and compartmentalized pools of CoA, which are crucial for the activation of carboxylate metabolites [19]. Given the high metabolic and signaling impact of certain acyl-CoA esters, precise control of their concentrations is essential. The carnitine system facilitates the transfer of activated acyl groups between cellular compartments, avoiding energy-expensive cycles of hydrolysis and resynthesis. Carnitine acyltransferases, such as CRAT, CPT1, CPT2, and CROT, play a central role in maintaining CoA homeostasis by converting acyl-CoA esters into membrane-permeable acyl-carnitine derivatives. This process prevents CoA sequestration, preserves free CoA pools, and protects cellular metabolism, as is evident in disease states where acyl group accumulation disrupts CoA homeostasis [19].

Structurally, CRAT belongs to the carnitine acyltransferase family, which includes the above-mentioned related enzymes (CPT1 and CPT2) and carnitine octanoyltransferase (CROT) [5,20,21,22,23]. These enzymes share a conserved acetyltransferase fold that facilitates substrate binding and catalysis, but they differ in substrate specificity and subcellular localization, enabling them to perform distinct yet complementary roles in lipid metabolism [5,20,21,22,23]. Mitochondrial CRAT, for example, predominantly mediates acetyl group transfer, while peroxisomal CROT and other members of the family are involved in the metabolism of medium- and long-chain acyl-CoA derivatives [5,20,21,22,23]. This compartmentalized enzymatic network underscores the complexity and versatility of carnitine acyltransferases in regulating cellular lipid metabolism and energy production.

The functional interplay between CRAT and the enzymes of the mitochondrial carnitine shuttle, together with the carnitine transporter, highlights CRAT’s role in regulating lipid metabolism. The carnitine shuttle system ensures the efficient transport of long-chain fatty acids into mitochondria for β-oxidation, fueling oxidative phosphorylation, with CPT1 catalyzing the initial conversion of acyl-CoA to acylcarnitine at the impermeable outer mitochondrial membrane [12,13,24,25,26,27,28]. The carnitine/acylcarnitine translocase (CACT, encoded by SLC25A20) facilitates the exchange of acylcarnitines and free carnitine across the highly permeable inner mitochondrial membrane, while CPT2 completes the cycle by regenerating acyl-CoA in the mitochondrial matrix for subsequent β-oxidation [12,13,24,25,26,27,28]. CRAT’s role in modulating acetyl-CoA levels complements this system, ensuring the efficient utilization of mitochondrial substrates and preventing metabolic bottlenecks [1,6]. In this regard, CRAT plays a crucial role in fatty acid metabolism, which depends on NADH and FAD cofactor availabilities [29]. The reduced cofactors can be re-oxidized through the respiratory chain complexes, flavoproteins, or with the support of the malate/aspartate shuttle [28,30,31,32], highlighting the pivotal role played by CRAT in lipid metabolism and energy production.

Beyond its canonical role in lipid metabolism, CRAT also influences amino acid catabolism and mitochondrial bioenergetics. Amino acid degradation produces acetyl-CoA either directly or through intermediates such as pyruvate and branched-chain keto acids, and CRAT’s activity ensures the proper handling of these acetyl groups to prevent mitochondrial dysfunction. Dysregulated CRAT activity has been implicated in several metabolic disorders, including obesity, insulin resistance, and mitochondrial diseases, highlighting its critical role in metabolic health.

Recent structural insights into CRAT have furthered our understanding of its enzymatic mechanism and regulation [20]. High-resolution X-ray crystallography and molecular modeling studies have elucidated the architecture of CRAT’s catalytic pocket, revealing key residues involved in substrate binding and catalysis. These findings have not only deepened our knowledge of CRAT’s structure–function relationship, but also paved the way for the development of small-molecule modulators aimed at restoring or enhancing its activity in disease contexts [33].

The therapeutic potential of targeting CRAT extends beyond rare genetic disorders to more common conditions characterized by metabolic dysfunction. Enhanced CRAT activity has been shown to improve mitochondrial efficiency and metabolic flexibility, suggesting its utility in addressing diseases such as diabetes and cardiovascular disorders [2]. Conversely, inhibiting CRAT activity may have applications in cancer therapy, where altered lipid metabolism supports tumor growth and proliferation [34,35,36]. Understanding the nuanced roles of CRAT and its interactions with carnitine shuttle components will be critical for developing targeted interventions that leverage its central role in metabolic regulation.

In this review, we provide a detailed exploration of CRAT, highlighting its multifaced roles in health and disease, with an emphasis on its structural and functional characteristics, its functional cooperation with the carnitine acyltransferases of the mitochondrial carnitine shuttle, and its potential as a therapeutic target. By integrating insights from enzymology, structural biology, and metabolic research, we aim to provide a comprehensive overview of CRAT’s critical contributions to metabolic homeostasis and its implications for future therapeutic strategies.

## 2. Functional Diversity of Carnitine Acyltransferases: Role of CRAT, CROT, CPT1, and CPT2 Across Cellular Compartments

The functional diversity of carnitine acyltransferases reflects the tissue and cell-compartment distribution of CRAT-like enzymes, such as CRAT, CROT, CPT1, and CPT2. While mitochondrial CRAT mediates the reversible transfer of acetyl groups between acetyl-CoA and carnitine, the peroxisomal CROT facilitates fatty acid oxidation of very long-chain fatty acids, which can enter peroxisomes thanks to the human peroxisomal fatty acid transporter ABCD1 [37]. The cytosolic enzyme CROT regulates the conversion of acyl(C4-C10)carnitines into acyl(C4-C10)-CoA, influencing metabolic adaptation.

**Figure 1:**
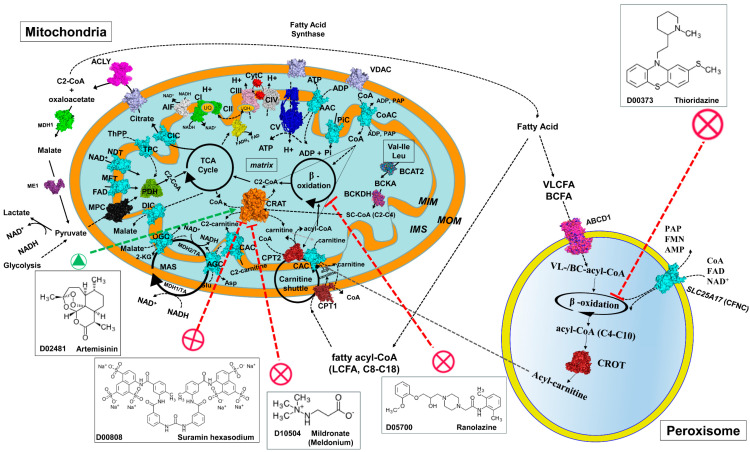
Scheme of the cross-talk between a mitochondrion and a peroxisome with a set of representative proteins, pathways and cycles. CRAT, CROT, protein of the carnitine shuttle, respiratory chain complexes, mitochondrial transporters and other proteins are reported in surf representation and labeled. CRAT (based on the human crystallized structure 1nm8.pdb) is reported in orange surface representation; the peroxisomal CROT (based on the human crystallized structure 1xl8.pdb) is reported in brown surface representation; the peroxisomal very long chain fatty acid (VLCFA) transporter ABCD1 (based on the crystallized structure 7rr9.pdb) is reported in magenta-blue surface representation; the mitochondrial branched chain amino acid aminotransferase BCAT2 (based on the crystallized structure 5mpr.pdb) is reported in blue-green surface representation; the mitochondrial branched chain alpha-ketoacid dehydrogenase BCKDH (based on the crystallized structure 1u5b.pdb) is reported in dark magenta-blue surface representation; CPT1 and CPT2 (both based on the *R. norvegicus* crystallized structure 4ep9.pdb) in dark violet; ATP synthase (CV) is reported in blue (based on the Bos taurus crystallized structure 6zqn.pdb). Mitochondrial carriers are reported in cyan (based on the 3D structure of the bovine ADP/ATP carrier, 1okc.pdb). VDAC is reported as a reference protein of the outer mitochondrial membrane in light-pink surface representation (based on the 3D structure of the human 2jk4.pdb). MPC (an in-house developed 3D comparative model, data not published) is reported in black; PDH in light green (based on the human crystallized structure 6cfo.pdb); AIF in white (based on the human crystallized structure 4bur.pdb). Complex I (CI, based on the *Ovis aries* crystallized structure 5lnk.pdb), complex II (CII, based on the *Sus scrofa* 3aef.pdb), complex III (CIII, based on the *O. aries* 6q9e.pdb), complex IV (CIV) (together with CytC in red, based on the bovine crystallized structure 5iy5.pdb) are reported in green, yellow, magenta and gray, respectively, according to PyMOL colors. Black circular arrows indicate cyclic pathways. Red arrows indicate impaired pathways or reactions. Black solid/dashed lines indicate the possible direction of the reported reactions. Abbreviations: C2-CoA, acetyl-CoA; C2-carnitine, acetyl-carnitine; SC-CoA, short chain acyl-CoA; LCFA, long chain fatty acids; VLCFA, very long chain fatty acids; BCFA, branched chain fatty acids; BCKA, branched chain ketoacids; MIM: mitochondrial inner membrane; MOM, mitochondrial outer membrane; IMS, intermembrane space; UQ, ubiquinone; CFNC, the peroxisomal CoA, FAD, NAD^+^/PAP, FMN, AMP carrier, coded in *H. sapiens* by SLC25A17; AAC, ADP/ATP carrier, coded in *H. sapiens* by SLC25A4, SLC25A5, SLC25A6, SLC25A31; TPC, thiamine pyrophosphate carrier, coded by SLC25A19; CAC, carnitine/acyl-carnitine carrier, coded by SLC25A20; ORC, ornithine carrier, coded by SLC25A15 (or SLC25A2); AGC, aspartate/glutamate carrier, coded by SLC25A12 and SLC25A13; DIC, dicarboxylate carrier, coded by SLC25A10; NDT, assumed to be the NAD+ carrier, coded by SLC25A51; MFT, assumed to be the FAD (folate/riboflavin) carrier, coded by SLC25A32; OGC, malate/2-oxoglutarate carrier, coded by SLC25A11; CIC, citrate carrier, coded by SLC25A1; PiC, phosphate carrier, coded by SLC25A3; CoAC, CoA carrier, coded by SLC25A42; MAS, malate/aspartate shuttle; TCA, tricarboxylic acid cycle; Bax, Bcl-2 associated X protein; Bak, Bcl-2 antagonist/killer-1; Bcl-2, B-cell lymphoma-2; MDH1, cytosolic malate dehydrogenase 1; ME1, malic enzyme 1; MPC, mitochondrial pyruvate carrier; PDH, pyruvate dehydrogenase; CypD, cyclophilin D; CytC, cytochrome C; VDAC, voltage-dependent anion channel; AIF, apoptosis-inducing factor; PNC, pyrimidine nucleotide carrier, coded in *H. sapiens* by SLC25A33 and SLC25A36. The green arrow for Artemisinin and the magenta arrow for suramin indicate the ability of artemisinin to stimulate the activity of p.Tyr110Cys variant and the ability of suramin to inhibit both the WT-CRAT and the p.Tyr110Cys variant. The other two magenta arrows for mildronate, ranolazine and thioridazine indicate their ability in partially inhibiting in a selective way CRAT, mitochondrial beta oxidation or peroxisomal beta oxidation, respectively. The reported 2D structures of the mentioned drugs have been collected from the KEGG-DRUG database (https://www.kegg.jp/kegg/drug/, accessed last time on the 23 January 2025). For all the mentioned drugs, the KEGG-DRUG identification number has been reported.

Westin et al. (2008) [5] highlighted how peroxisomal enzymes like CROT, in combination with acyl-CoA thioesterases (ACOT5, for medium chain thioesterase and ACOT12, for short chain thioesterase), work together to export β-oxidation products out of peroxisomes. These enzymes facilitate the movement of acylcarnitines into the cytosol for further metabolization or transport, ensuring proper lipid balance across compartments and contributing to cellular homeostasis (Figure 1). This peroxisomal system complements the mitochondrial carnitine shuttle, allowing for efficient lipid metabolism in different cellular contexts [5].

Remarkably, beyond the well-characterized mitochondrial CRAT and peroxisomal CROT, Westin et al. [5], and Ramsay et al. [4] also proposed the existence of a CRAT-like enzyme (carnitine short-chain acyltransferase) in the cytosol and in peroxisomes. Conversely, more recent models from Lasheras-Otero et al. [23] proposed the existence of an extra CRAT-like enzyme only in peroxisome, whereas Hsu et al. proposed the existence of an extra CRAT-like enzyme only in the cytosol [3]. More generally, this extra carnitine acyltransferase activities deserve dedicated studies for validating the localization of CRAT-like enzyme isoforms out of mitochondria and peroxisome.

Concerning the links between the mitochondrial CRAT and the carnitine shuttle, in mitochondria, CPT1 catalyzes the conversion of long-chain acyl-CoA into acylcarnitines, enabling their transport into the mitochondrial matrix via the carnitine/acylcarnitine translocase (SLC25A20) [13,26,38,39]. Once inside, CPT2 regenerates acyl-CoA for β-oxidation [1,23,24]. All of these enzymes, together with acyl-CoA thioesterases (i.e., ACOT5 and ACOT12), create a tightly coordinated system to manage lipid metabolism and energy production and illustrate the compartmentalized nature of lipid metabolism, ensuring fatty acids are properly shuttled and oxidized across cellular compartments (Figure 1).

Remarkably, selective inhibitors of mitochondrial β-oxidation (i.e., ranolazine) and peroxisomal β-oxidation (i.e., thioridazine) were investigated for their ability to regulate the pool of fatty acids with important implications for the treatment of cardiovascular diseases [24,40], or neurodegeneration with an impaired mitochondrial function [25,41] (Figure 1). Similarly, mildronate was proposed as a CRAT inhibitor with cardioprotective properties, likely mediated by CRAT inhibition and the concomitant regulation of cellular energy metabolism, above all under ischemic conditions [42] (Figure 1). It was also proposed that mildronate can inhibit γ-butyrobetaine hydroxylase [43] or impair carnitine transport, in the context of lipid metabolism [44]. Among the ligands structurally related to carnitine, DL-aminocarnitine and acetyl-DL-aminocarnitine deserve to be mentioned for their abilities in modulating the activity of the mentioned carnitine acyltransferases, with therapeutic implications for the regulation of lipid metabolism [45]. More generally, emerging research underscores the interplay between the mentioned acyltransferases in maintaining metabolic equilibrium. Dysregulation of their activity has been linked to metabolic diseases, such as obesity, cardiovascular disorders, and certain cancers, highlighting their significance as potential therapeutic targets [6,46,47].

## 3. Structural Similarities Between Carnitine Short-/Medium-Long Acyltransferases and Choline Acetyltransferase: Active Site Architecture and Substrate Specificity

From a structural point of view, the crystallized CRAT, CROT, and CPT2 show a very similar structural architecture, with a root mean square deviation (RMSD) of their atomic coordinates ranging between 1.21 and 1.6 Å (Table 1) and a % of identical residues ranging between 19 and 39% (Table 2) [1,22,33,48,49].

Remarkably, another acyltransferase with an overall structure very similar to the structure of the above-mentioned carnitine acyltransferases is represented by the human choline acetyltransferase (PDB-ID: 2fy3.pdb [50]) showing a RMSD and % of identical residues with CRAT comparable to those observed among carnitine acetyltransferases [1,22,33,48,49] (Table 1 and Table 2). Choline acyltransferase (ChAT), while functionally distinct, shares structural similarities with carnitine acyltransferases. Both enzyme families belong to the larger family of transferases and exhibit comparable domain architectures.

These enzymes typically exhibit a two-domain structure comprising an N-terminal domain and a C-terminal domain. The N-terminal domain consists of an eight-stranded β-sheet flanked by α-helices, while the C-terminal domain features a six-stranded mixed β-sheet accompanied by multiple α-helices. This structural organization is crucial for their catalytic function and substrate specificity [1,22,33,48,49].

The active sites of these enzymes are strategically located at the interface between the N and C domains, with a histidine residue playing a pivotal role in catalysis. In the human CRAT (1nm8.pdb, complexed with carnitine and CoA, docked in 1nm8.pdb after superimposition operations [1,33] with the *Mus musculus* Crat, 1s5o.pdb [49]), for instance, His343 is identified as the catalytic residue. This histidine is accessible via two channels that allow substrate entry: one for carnitine, and another for Coenzyme A (CoA). The side chain of His343 forms a hydrogen bond with the carbonyl oxygen of the substrate, facilitating the transfer of acyl groups [1,22,33].

In ChAT, the active site also features a histidine residue that is critical for its catalytic mechanism. Structural studies suggest that choline binds in a position analogous to carnitine in CRAT, with the catalytic histidine facilitating the transfer of the acetyl group to choline, forming acetylcholine [49,51].

The binding sites for carnitine and CoA in the mentioned acyltransferases are characterized by specific residues that ensure proper substrate orientation and stabilization. In CRAT, the CoA binding site involves interactions with Lys419 and Lys423, which engage with the 3′-phosphate of CoA. Additionally, Asp430 and Glu453 form a hydrogen bond that is essential for maintaining the structural integrity of the binding site.

Carnitine binding involves the formation of a hydrogen bond between the 3-hydroxyl group of carnitine and the ε2 nitrogen of the catalytic histidine. This interaction is stereospecific, as only the correct isomer of carnitine can effectively bind and undergo catalysis. The binding site is composed of elements from both the N and C domains, creating a pocket that accommodates carnitine’s structure. Upon binding, carnitine is partially exposed to the solvent, which may facilitate product release after the catalytic reaction [1,22,33].

**Figure 2:**
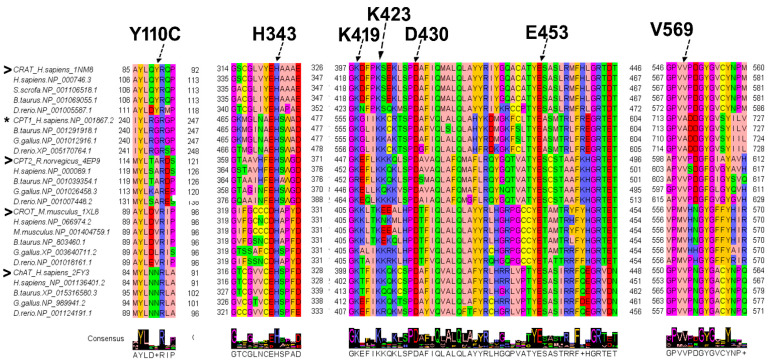
Multiple sequence alignment of the human carnitine acyltransferases and choline acyltransferase. An extract from a multiple sequence alignment of carnitine acyltransferases and choline acetyltransferase sampled by blastp through the indicated metazoan is reported. The represented blocks highlight the conservation of residues crucial for the function of the compared enzymes, such as H343 (important for the catalytic activity), K419 and K23 (important for CoA binding), and D430 and E454 (important for protein stability). All the mentioned residues are labeled and indicated by arrows. Amino acid replacement Y110C and V569M recently associated with an early onset case of Leigh syndrome are also reported for comparative purposes and indicated by labels and arrows. Residue numbering in the amino acid labels corresponds to the residues numbering in the human CRAT sequence with accession number NP_000746.3. The “>” symbols indicate the crystallized carnitine acyltransferases or the choline acetyl transferase, reported in this alignment for comparative purposes, with reference to residues numbering that can vary between the crystallized structures and sequences retrieved by blastp (see also Table 1). The “*” symbol indicates CPT1 that has not a crystallized counterpart. The alignment was obtained by using ClustalW 2.1 (a free software currently maintained at the Conway Institute UCD Dublin by Des Higgins, Fabian Sievers, David Dineen, and Andreas Wilm) in the Jalview 2.11.4.1 package (a free software released under GPLv3, developed by The Barton Group, University of Dundee, Scotland UK). Amino acid colors reflect their physico-chemical properties according the Jalview “zappo” color scheme: aliphatic/hydrophobic residues Alanine (A), Isoleucine (I), Leucine (L), Methionine(M), Valine (V) are colored in light-pink; aromatic residues Phenylalanine (F), Tryptophan (W), Tyrosine (Y) are colored in orange; conformationally special Gycine (G) and Proline (P) are colored in magenta; Cysteine (C) is colored in yellow; hydrophilic residues Asparagine (N), Glutamine (Q), Serine (S), Threonine (T) are colored in green; acidic negatively charged residues, Aspartate (D), Glutamate (E) are colored in red; basic positively charged residues Arginine (R), Histidine (H), Lysine (K) are colored in blue [55,56].

**Figure 3:**
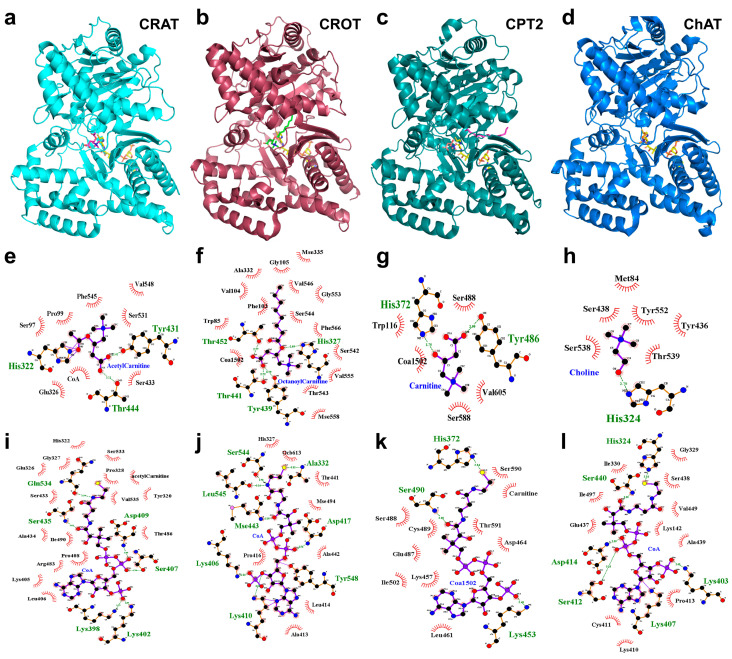
Three-dimensional comparative analysis of the human carnitine acyltransferases and choline acyltransferase. The crystallized structures of CRAT, CROT, CPT2, and ChAT are reported in cyan, dark-pink, dark-green, and blue cartoon representations (by PyMOL [63]) in panels (**a**–**d**), respectively. CoA is reported in yellow sticks in all the a-d panels. Acetylcarnitine is reported in magenta sticks in panel (**a**), octanoylcarnitine is reported in green sticks in panel (**b**), carnitine is reported in salmon sticks in panel (**c**), whereas choline is reported in orange sticks in panel (**d**). The binding sites of acetylcarnitine, octanoylcarnitine, carnitine, and choline in the four crystallized structures are reported in panels (**e**–**h**), whereas CoA binding sites are reported in panels (**i**–**l**). The ligands and protein side chains are shown in ball-and-stick representation, with the ligand bonds colored in purple, according to the default style of LigPlot [64]. Hydrogen bonds are shown as green dotted lines, while the spoked arcs represent protein residues making nonbonded contacts with the ligand. Residues numbering in panels (**e**) and (**i**) reflects the numbering of the crystallized structure of CRAT lacking 21 residues at the N-terminal., i.e., the real residues numbering of CRAT residues is obtained by adding 21 residues (to be considered for comparative purposes with reference to residues numbering reported in the multiple sequence alignment).

It should be noted that, despite the relatively low percentage of identical residues (between 19 and 40%) the multiple sequence alignment of the mentioned carnitine acyl transferases shows conserved residues in correspondence of the CRAT H343, K419, K423, D430, and E453 (Figure 2). H343 residue in the CRAT active site acts as a general base in the carnitine acetyltransferase activity [4,20,52]. H343 extracts the proton from the 3-hydroxyl group of carnitine or the thiol group of CoA, depending on the direction of the reaction. Notably, the CoA binding site is on the opposite side of the H343 side chain with reference to the carnitine binding site [4,20,52]. Among crucial residues contributing to and involved in CoA binding, K419 (K398 in Figure 3i) and K423 (K402 in Figure 3i) are conserved among all of the mentioned carnitine acyltransferases (Figure 2 and Figure 3i–l). Both lysine residues recognize the 3′-phosphate group of CoA (Figure 3i–l) [4,20,52]. In the binding site for the pantothenic arm of CoA, D430 and E453 (also conserved in the other analyzed carnitine acyltransferases) are directly hydrogen-bonded to each other contributing to the proper orientation of substrates (above all D430) within the catalytic pocket, and participating to the structural integrity of the active site. Mutation of either residue can reduce the activity of the enzyme [4,20,52]. Notably, while the mentioned residues are conserved across all compared carnitine acyltransferases, they are situated within protein regions that exhibit significant variability among the different enzyme groups. The variability in amino acid composition within the reported blocks reflects the substrate specificity of the analyzed enzymes with reference to their abilities in processing short, medium and long chain acyl-moieties.

The aligned residues occupy also the same position in the space around substrates and cofactors of the investigated acyltransferases, as observed in the corresponding 3D crystallized structures of CROT (1xl8.pdb, complexed with octanoylcarnitine, and CoA, being the latter docked in 1xl8.pdb [53], after superimposition operations with the *Mus musculus* Crat enzyme, 1s5o.pdb, previously described [1]), the human ChAT (2fy3.pdb, complexed with choline and CoA, being the latter docked in 2fy3.pdb after superimposition operations with another ChAT crystallized structure, 2fy4.pdb [50]), the *Rattus norvegicus* CPT2 (2deb.pdb, complexed with CoA and palmitate [54]) (Figure 3). Mutations in these residues can lead to a significant decrease in enzymatic activity, underscoring their importance in the catalytic process [1,22,33].

Regarding substrate specificity, CRAT prefers short-chain (C2–C4) acyl-CoAs. In contrast, CROT prefers medium-chain (C4–C10) acyl-CoAs, such as octanoyl-CoA, while CPT1 and CPT2 are specific for long-chain (C8–C18) fatty acyl-CoAs, playing a crucial role in the transport and β-oxidation of long-chain fatty acids in mitochondria. These substrate preferences are dictated by the structural composition of their acyl-binding sites, where specific residues create an environment conducive to binding acyl groups of particular chain lengths [1,22,33]. Table 3 reports the Km values for various acyl-CoA derivatives, carnitine, acyl-carnitine derivatives, and CoA, as determined under different experimental conditions (e.g., proteins purified from animal tissues, yeast, or recombinant proteins expressed in bacteria) for the reviewed CRAT, CROT, CPT1, and CPT2 enzymes.

**Table 3:** Km Values for CRAT, CROT, CPT1, CPT2, and ChAT with various Acyl-CoA substrates, Acyl-Carnitine substrates, CoA, and carnitine. The reported Km values are as presented in the cited papers, where data on Vmax and other kinetic parameters can also be found. (# Rat liver; § Rat skeletal muscle; * purified mitochondria from animal tissues).

	CRAT	CROT	CPT1	CPT2	
	K_m_ (μM)	K_m_ (μM)	K_m_ (μM)	K_m_ (μM)	
	58.0 ± 9.6				[1]
	20.39 ± 8.77				[33]
Acetyl-CoA	25	77			[3]
	34				[57] *
	240 ± 47	78 ± 18			[58]
Acetyl-3′-dP-CoA	1300				[57] *
	120				[57,59] *
L-Carnitine	120	108	30 ^#^ (500 ^§^)	1500	[4] *
		172 ± 46	127 ± 4		[60]
			500 ^§^		[61] *
Acetyl-L-carnitine	350				[57,59] *
CoA	37				[59] *
	37	7.4	40 #		[4] *
Propionyl-CoA	36.0 ± 3.4				[1]
	50	49			[3]
Butyryl-CoA	499 ± 44				[58]
	58	41			[3]
Hexanoyl-CoA	55	60			[3]
Octanoyl-CoA	95.4 ± 3.4				[1]
	21	24			[3]
*Trans*-2-C4:1-CoA	590 ± 129				[58]
Decanoyl-CoA		2.0 ± 0.2	4.9 ± 0.3		[60]
Myristoyl-CoA			28.2 ^#^		[61] *
Palmitoyl-CoA				7.1	[62]
Palmitoleoyl-CoA				8.1	[62]
Acyl-CoA	34	34		<5	[4] *
Acyl-carnitine	350	7.4		46	[4] *

In summary, carnitine acyltransferases and choline acetyltransferase share a conserved structural framework that includes a two-domain architecture and a catalytic histidine residue. The specific residues within their binding sites determine substrate specificity and catalytic efficiency, enabling these enzymes to perform their essential roles in cellular metabolism.

## 4. Functional and Structural Implications of the Crosstalk Between Carnitine Acyltransferases and Carnitine/Acylcarnitine Transporter

Considering what was reported above, it is evident that CRAT (together with other acyltransferases structurally related to CRAT, such as CROT, CPT1 and CPT2) serves multiple physiological functions:

- Regulation of Acetyl-CoA Levels: by converting excess acetyl-CoA into acetylcarnitine, CRAT helps maintain acetyl-CoA homeostasis, preventing the accumulation that could disrupt metabolic processes [1,2,3,4,5,23,65].
- Modulation of cellular energy metabolism: the interconversion between acetyl-CoA and acetylcarnitine allows for the buffering of acetyl groups, influencing energy production pathways [1,2,3,4,5,23,66].
- Epigenetic regulation: recent studies suggest that acetylcarnitine shuttling links mitochondrial metabolism to histone acetylation, thereby influencing gene expression [1,2,3,4,5,23,46].

In addition, the mitochondrial CAC, encoded by the SLC25A20 gene, also plays a crucial role in fatty acid metabolism, facilitating the transport of acylcarnitines across the inner mitochondrial membrane in exchange for free carnitine. This transport is essential for the β-oxidation of fatty acids within the mitochondrial matrix, a primary energy source, especially during periods of fasting or increased energy demand.

Structurally, CAC is a member of the mitochondrial carrier family (SLC25A) and plays a pivotal role in the mitochondrial carnitine shuttle, ensuring efficient fatty acid oxidation [67]. Recent studies by Giangregorio et al. have uncovered details of the transient dimerization of the carnitine/acylcarnitine carrier, suggesting a complex mechanism behind its functional regulation [26]. Dimerization may influence carrier activity, highlighting its critical role in maintaining the efficiency of fatty acid transport across mitochondrial membranes.

More generally, mitochondrial carriers shuttle metabolites (substrates and cofactors) crucial for cell viability across the inner mitochondrial membrane, and mutations occurring at several mitochondrial carriers, including CAC, were associated with severe mitochondrial diseases [24,25,68,69,70,71,72,73,74].

Mitochondrial transporters of the SLC25A family are characterized by three homologous domains, each comprising two transmembrane α-helices [75,76,77]. This configuration forms a central cavity that accommodates substrate binding and translocation. Despite extensive research, the high-resolution 3D structure of CAC and most of mitochondrial carriers remains undetermined, posing challenges to a comprehensive understanding of its transport mechanism [13,26,76,77]. Nonetheless, functional studies have elucidated that CAC operates via an antiport mechanism, exchanging acylcarnitine from the cytosol with carnitine from the mitochondrial matrix [13,26]. This exchange is vital for maintaining the mitochondrial acyl-CoA pool necessary for β-oxidation.

CAC-mediated transport ensures the availability of acyl groups within the mitochondrial matrix for β-oxidation, while CRAT-mediated acetyl group transfer regulates acetyl-CoA levels, influencing both energy production and biosynthetic pathways. In the homeostasis of acetyl-CoA pool, the cytosolic ATP-citrate lyase (ACLY) plays also an important role [78,79]. Disruptions in the function of each of the mentioned proteins can lead to metabolic imbalances [1,73,78,79].

Mutations in the SLC25A20 gene can result in carnitine-acylcarnitine translocase deficiency, a condition characterized by impaired fatty acid oxidation. Clinically, this manifests as hypoketotic hypoglycaemia, cardiomyopathy, muscle weakness and, in severe cases, sudden infant death. Early diagnosis and management, including dietary modifications and carnitine supplementation, are essential for affected individuals [24,25,73].

Understanding the structural and functional dynamics of CAC and CRAT is not only fundamental research in biochemistry, but also has clinical implications and the coordinated actions of CAC, CRAT, CROT and other enzymes of the carnitine shuttle are central to cellular energy metabolism, facilitating the efficient transport and utilization of fatty acids within mitochondria. Ongoing research continues to uncover the complexities of their functions and interactions, offering insights into metabolic regulation and potential therapeutic avenues for metabolic diseases.

## 5. CRAT and Acylcarnitine-Mediated Epigenetic Regulation of Branched-Chain Amino Acid and Lipid Metabolism

CRAT and its role in epigenetic regulation have emerged as key areas of interest in understanding cellular metabolism. Indeed, it has been proposed that CRAT modulates the intracellular acetyl-CoA pool and links mitochondrial energy metabolism to epigenetic mechanisms, particularly histone acetylation—a key epigenetic modification that can influence gene expression patterns related to metabolism [1,46].

Furthermore, the carnitine system has been identified as a critical component in cellular metabolic plasticity, particularly in cancer cells [11]. Indeed, it was highlighted that the carnitine system plays an important role in modulating metabolic pathways, including lipid metabolism, through epigenetic mechanisms [11]. By influencing the availability of acyl groups for histone modification, carnitine acyltransferases can affect the expression of genes involved in lipid biosynthesis and degradation, thereby altering cellular lipid profiles [11].

Remarkably, it was observed that elevated CRAT and CROT expression are linked to enhanced metastatic potential in melanoma, emphasizing their role as regulators of mitochondrial and peroxisomal cross-talk for the handling of acylcarnitine shuttle, which can promote metastasis [3,23]. These enzymes work in concert with the mitochondrial CAC and other acyltransferases to regulate lipid balance, providing cellular flexibility in response to varying metabolic demands [3,23,24,25]. The integration of these systems is critical for overall energy metabolism, and dysfunction in any of these enzymes can lead to metabolic disturbances, as seen in several diseases related to mitochondrial and peroxisomal dysfunction [1,24,25,33,73,80,81].

In the context of BCAA metabolism, carnitine acyltransferases facilitate the transport and subsequent oxidation of branched-chain acyl-CoA derivatives (Figure 1). Disruptions in this system can lead to the accumulation of BCAA metabolites, which have been implicated in metabolic disorders [82,83,84]. Epigenetic modifications, such as DNA methylation and histone acetylation, play roles in the regulation of genes involved in BCAA catabolism. By modulating the intracellular concentrations of acyl-CoA and acetyl-CoA, carnitine acyltransferases indirectly influence these epigenetic marks, thereby affecting the expression of metabolic genes. Amino acid catabolism generates acetyl-CoA either directly or via intermediates such as pyruvate and branched-chain keto acids.

Recent findings highlight the interplay between amino acid metabolism and lipid homeostasis in the context of cancer [82,84]. Specifically, branched-chain amino acid (BCAA) catabolism influences fatty acid import into mitochondria by modulating the levels of acetyl-CoA and malonyl-CoA, which are key regulators of carnitine palmitoyltransferase 1 (CPT1).

Disruption of this pathway in pancreatic cancer cells redirects fatty acids toward triglyceride synthesis and storage, thereby altering cellular lipid dynamics. CRAT’s role in managing mitochondrial acetyl-CoA levels positions it as a central player in this metabolic axis, ensuring efficient carbon flux and preventing metabolic overload.

In health, CRAT supports metabolic flexibility by buffering mitochondrial acetyl-CoA levels [1], maintaining optimal conditions for continued amino acid oxidation. However, in pathological states such as cancer and metabolic disorders, dysregulation of CRAT activity contributes to impaired amino acid and lipid metabolism, potentially driving metabolic inflexibility and disease progression. The regulatory role of CRAT in amino acid metabolism underscores its potential as a therapeutic target for disorders characterized by disrupted mitochondrial and metabolic function [82,84].

By controlling the availability of substrates for both metabolic reactions and histone modifications, the carnitine carrier indirectly influences epigenetic regulation of gene expression. Alterations in carnitine transport can thus have downstream effects on lipid and BCAA metabolism through epigenetic mechanisms [1,82,84].

In summary, CRAT and related carnitine acyltransferases, along with the carnitine carrier, serve as crucial links between cellular metabolism and epigenetic regulation. By modulating the availability of acyl-CoA and acetyl-CoA, these components influence histone modifications and, consequently, the expression of genes involved in lipid and BCAA metabolism.

## 6. The Role of CRAT in Acetyl-CoA Homeostasis and Metabolic Flexibility in Health and Disease

It has also been proposed that CRAT enzymatic function facilitates the mitochondrial efflux of acetyl moieties, alleviating the inhibitory effects of excess acetyl-CoA on critical metabolic processes such as pyruvate dehydrogenase (PDH) activity [6,85].

Studies in muscle-specific CRAT knockout mice reveal that CRAT deficiency disrupts mitochondrial carbon flux, leading to metabolic inflexibility characterized by impaired substrate switching between glucose and fatty acids. This disruption exacerbates glucose intolerance and enhances susceptibility to diet-induced insulin resistance, as excess acetyl-CoA accumulates, fueling lysine hyperacetylation of mitochondrial proteins and potentially inhibiting respiratory chain complexes [1,2].

Furthermore, altered CRAT activity influences systemic acetyl-carnitine levels, which are implicated in neuroprotection and metabolic regulation [1]. The dynamic equilibrium of acetyl-CoA and acetyl-carnitine facilitated by CRAT is essential for metabolic flexibility, as it buffers mitochondrial acetyl-CoA levels, thereby modulating PDH activity and enabling efficient transitions from fatty acid to glucose oxidation during metabolic shifts [1,2,6,85]. In disease states such as diabetes and mitochondrial disorders, dysregulated CRAT activity contributes to metabolic dysfunction, underscoring its therapeutic potential.

## 7. CRAT: A Key Regulator of Lipid Metabolism and Mitochondrial Carbon Flux

The interconversion of short-chain acyl-CoAs in their corresponding acylcarnitines mediated by CRAT facilitates the homeostasis of acetyl groups between mitochondria and cytosol, preserving mitochondrial function and preventing metabolic overload [1]. In the context of fatty acid β-oxidation, CRAT ensures efficient carbon flux by converting acetyl-CoA, a byproduct of fatty acid degradation, into acetyl-carnitine, which can contribute to systemic energy metabolism.

Studies indicate that CRAT expression is upregulated during calorie restriction (CR), leading to increased acetyl-carnitine levels and enhanced metabolic flexibility [6]. This adaptation supports mitochondrial energy production and modulates lipid-derived acetyl-CoA flux, preventing its accumulation and the associated inhibitory effects on PDH [1,2,6,85]. Furthermore, CRAT activity appears to coordinate with the carnitine shuttle system, ensuring the balanced transport of long-chain fatty acids for β-oxidation and the removal of their metabolic byproducts.

Dysregulation of CRAT or its interaction with lipid metabolic pathways has been implicated in conditions of metabolic inflexibility, such as obesity and insulin resistance, highlighting its therapeutic potential for metabolic disorders [2]. Emerging evidence underscores the role of the mitochondrial CRAT in modulating the lipid-acetyl-CoA pool together with the peroxisomal CROT, the members of the carnitine shuttle (CAC, CPT1 and CPT2) and acyl-CoA thioesterases, maintaining the equilibrium between fatty acid oxidation and systemic energy balance [10] (Figure 1).

## 8. Role of CRAT in Lung and Heart Function: Implications for Cellular Lipid Metabolism and Immune Regulation

CRAT is increasingly recognized for its critical involvement in lipid metabolism and immune responses within the lung and heart. In pulmonary cells, CRAT regulates key metabolic pathways that influence nitric oxide (NO) signaling, a crucial mediator of vascular tone and endothelial cell function [8]. As nitric oxide production is tightly linked to cellular energy status and lipid metabolism, CRAT plays a dual role in modulating these pathways, thus influencing both pulmonary vascular health and immune responses. In particular, CRAT-mediated acetylation of carnitine facilitates the transfer of acetyl groups across mitochondrial membranes, optimizing fatty acid metabolism and contributing to the maintenance of endothelial cell homeostasis. Recent studies have shown that dysregulation of CRAT activity in the lungs can exacerbate pulmonary arterial hypertension and contribute to endothelial dysfunction, highlighting the enzyme’s essential role in maintaining cardiovascular and pulmonary integrity under stress conditions [47,86].

Similarly, in the heart, CRAT has emerged as a critical modulator of lipid metabolism and innate immune responses. Research has shown that CRAT links cholesterol metabolism to the regulation of inflammatory pathways, with significant implications for cardiovascular health [47]. In particular, CRAT affects the availability of acetyl-CoA for lipid biosynthesis, influencing the lipid composition of cell membranes and modulating immune cell activation in the myocardium [6,47]. The enzyme’s ability to modulate innate immune signaling pathways, especially in response to stress or injury, underscores its potential role in inflammatory heart diseases [87]. In recent work, it was demonstrated that CRAT regulates macrophage activity and cytokine release, suggesting that CRAT’s impact extends beyond metabolic regulation to immune function, influencing heart inflammation and contributing to the progression of conditions such as atherosclerosis and myocardial infarction [47,86,88].

Moreover, the crosstalk between CRAT activity and lipid metabolism in both pulmonary and cardiac tissues extends to broader metabolic disturbances. For instance, altered CRAT expression can shift the balance of lipid intermediates within immune cells, exacerbating inflammatory responses and promoting metabolic diseases like obesity and diabetes [6]. This connection between lipid metabolism and immune regulation is particularly crucial in chronic heart and lung diseases [24,89,90,91], where inflammation and metabolic dysfunction are often intertwined. As such, targeting CRAT activity offers therapeutic potential in modulating both lipid metabolism and immune responses in these tissues [24,89,90,91,92]. By enhancing or inhibiting CRAT’s function, it may be possible to restore balance in lipid and inflammatory pathways, potentially offering novel interventions for a range of cardiovascular and pulmonary disorders.

In conclusion, CRAT serves as a crucial nexus between lipid metabolism and immune regulation in both the heart and lungs. Its dual role in regulating metabolic processes and immune responses makes it an attractive target for therapeutic strategies aimed at treating diseases where both metabolic dysfunction and inflammation play a significant role. Understanding the precise mechanisms by which CRAT influences these pathways could lead to more effective treatments for a variety of cardiovascular and pulmonary conditions, offering promise for better management of diseases such as pulmonary arterial hypertension, heart failure, and atherosclerosis.

## 9. Structural and Functional Insights into Carnitine O-Acetyltransferase (CRAT): Therapeutic Modulation and Its Potential for Treating Mitochondrial Diseases and in Cancer Therapy

Recent structural studies have focused on understanding CRAT enzyme mechanisms, particularly through the examination of genetic variants like p.Tyr110Cys, which have been linked to mitochondrial diseases, such as Leigh syndrome. This variant results in a dysfunctional CRAT enzyme, impacting mitochondrial bioenergetics and contributing to early-onset neurodegenerative conditions [1,33], and concerns a residue highly conserved in CRAT orthologs (see supp. info of [1]), but not in the other human carnitine acyltransferases or in choline acyltransferase (Figure 2).

The structural basis for CRAT’s function and its variants is increasingly understood through high-resolution techniques such as X-ray crystallography and computational modeling. For instance, in silico studies, including molecular docking simulations, have identified several potential small molecules capable of modulating CRAT activity. In the case of the p.Tyr110Cys variant, associated with Leigh syndrome [1], these in silico approaches have been combined with in vitro assays to discover modulators that could either restore or inhibit CRAT activity, offering promising therapeutic avenues. Notably, one of the two bioactive small molecules selected through the virtual screening of chemical libraries, namely artemisinin, was found to stimulate the activity of the CRAT mutant, suggesting a potential route for therapeutic intervention in CRAT-related mitochondrial disorders [1,33].

Therapeutically, CRAT modulation could provide a means to treat not only genetic disorders like Leigh syndrome, but also other conditions related to mitochondrial dysfunction. The development of selective CRAT modulators, through the study of the structure–function relationships of the enzyme, holds promise for more targeted interventions with fewer side effects. Furthermore, exploring how CRAT interacts with mitochondrial networks and influences overall metabolic pathways can facilitate the development of strategies to combat disorders beyond those caused by genetic mutations, such as those involving age-related mitochondrial decline, metabolic syndromes and specific cancer types.

Notably, suramin, a bioactive compound recently proposed as a CRAT inhibitor [33], has been shown to be able to regulate potentially the acetyl-CoA/acetyl-carnitine pools at low concentrations (Figure 1). This regulation can be particularly relevant in inflammatory diseases and some cancer types exhibiting mitochondrial dysfunction including defects in the respiratory chain or fatty acid translocation and metabolism [24,25,78,79,93,94].

However, suramin is known to interact with multiple proteins, including the ADP/ATP carrier (inhibited to a greater extent than other carriers [75]), succinate dehydrogenase [39], or purine and peptide receptors [40], yet it is not listed in any PAINS database [95].

Interestingly, many of suramin’s targets, such as the ADP/ATP carrier, succinate dehydrogenase (FAD-dependent), purine receptors, and CRAT (CoA-dependent), interact with substrates or cofactors containing purine rings, letting us hypothesize that suramin targets purine binding regions in all the mentioned enzymes and transporters [33]. This hypothesis, combined with suramin’s intrinsic chemical and physical properties (such as flexibility and charged functional groups [96]), may explain suramin ability to efficiently target multiple proteins.

In this regard, suramin’s potential repurposing as a cancer therapeutic appears promising, provided that its effective concentrations for targeting CRAT, the ADP/ATP carrier, succinate dehydrogenase, and purine receptors [33], often dysregulated in specific oxidative cancers [12,83,97,98,99,100], can be achieved at doses lower than those historically used to treat other conditions, ranging from neglected tropical diseases to cancer [96].

## 10. Conclusions

CRAT and related enzymes play central roles in metabolic regulation by maintaining acetyl-CoA homeostasis, supporting metabolic flexibility, and facilitating lipid and amino acid metabolism. Through their activity across mitochondrial, cytosolic, and peroxisomal compartments, these enzymes ensure efficient substrate utilization and energy production. CRAT’s function in modulating acetyl-carnitine levels connects energy metabolism with epigenetic regulation, further influencing cellular adaptation and gene expression.

The enzymes involved in carnitine/acylcarnitine metabolism hold significant potential as therapeutic targets, particularly in the context of mitochondrial diseases and cancer. Mitochondrial dysfunction, which is often characterized by impaired fatty acid oxidation and energy production, can be mitigated by modulating the activity of mitochondrial carnitine/acylcarnitine carrier, CRAT, CROT, and other acetyltransferases structurally related to CRAT, aiming to restore normal lipid transport and energy metabolism in cells affected by mitochondrial diseases.

Recent studies [24,25,101,102] have explored pharmacological approaches to enhance mitochondrial function, focusing on compounds that can optimize the activity of carnitine acyltransferases. For example, nutritional supplements and their analogs, such as acetyl-L-carnitine, have shown promise in ameliorating mitochondrial dysfunction associated with cardiovascular diseases and other mitochondrial disorders. These compounds may help by increasing the availability of carnitine derivatives, thus improving fatty acid oxidation and mitochondrial energy production [24,101,102,103]. Additionally, the exploration of targeted therapies that modulate carnitine acyltransferase activity could also be beneficial in treating metabolic diseases linked to dysfunctional fatty acid metabolism.

In cancer therapy, the CRAT family’s involvement in lipid metabolism has opened new avenues for therapeutic development. Altered lipid metabolism is a hallmark of cancer cells, with increased fatty acid oxidation supporting the energetic and biosynthetic demands of rapidly proliferating tumors. In this context, targeting enzymes like CROT, CRAT, CPT2, CPT1, and CAC, which regulate lipid transport and utilization, may inhibit cancer cell growth by disrupting their metabolic flexibility. Recent research on the dynamic behavior of the carnitine carrier underscores the importance of pathway led by CAC and carnitine-dependent acyltransferases in cancer cell metabolism, suggesting that modulation of this system could become a novel strategy for cancer treatment, aiming at reprogramming metabolism in mitochondrial diseases and cancer, providing a multifaceted approach to treatment development.

In particular, CRAT represents an underestimated promising target for addressing metabolic disorders such as obesity, diabetes, mitochondrial diseases, and certain cancers. Enhancing CRAT activity could restore metabolic equilibrium in conditions of mitochondrial dysfunction, while inhibiting its activity may provide opportunities for disrupting cancer cell metabolism. Advances in structural biology and pharmacological research, through the combined use of computational tools and experimental validation, as exemplified by recent studies [33], offer an efficient approach to drug discovery, opening avenues for new therapeutic modalities targeting CRAT’s enzyme activity, and paving the way for precision medicine approaches.

Future research should prioritize understanding the detailed regulatory mechanisms governing CRAT and its interactions with other metabolic pathways. Investigating genetic and environmental factors that influence its activity will enable the development of targeted interventions for metabolic and proliferative diseases, ultimately improving patient outcomes.

## Figures and Tables

**Table 1:** RMSD matrix of residue atomic coordinates comparing human carnitine acyltransferases and choline acetyltransferase.

RMSD (Å)	CRAT (1nm8.pdb)	ChAT (2fy3.pdb)	CROT (1xl8.pdb)	CPT2 (4ep9.pdb)
**CRAT_NP_000746.3**	0	1.29	1.21	1.35
**ChAT_NP_001136401.2**		0	1.49	1.39
**CROT_NP_066974.2**			0	1.6
**CPT2_NP_000089.1**				0

**Table 2:** Matrix of the percentage of identical residues comparing human carnitine acyltransferases and choline acetyltransferase.

% Identical Residues	CRAT	ChAT	CROT	CPT1	mtCPT2
**CRAT_NP_000746.3**	ID	39.3	30.6	22.2	26.2
**ChAT_NP_001136401.2**		ID	28	22.5	24.7
**CROT_NP_066974.2**			ID	20.9	24.5
**CPT1_NP_001867.2**				ID	19.4
**CPT2_NP_000089.1**					ID

## Data Availability

The data that support the findings of this study are available from the corresponding author upon reasonable request. Data will be made available on request.
